# A cross-sectional study of the epidemic situation on COVID-19 in Gansu Province, China – a big data analysis of the national health information platform

**DOI:** 10.1186/s12879-020-05743-8

**Published:** 2021-02-05

**Authors:** Xuanchen Yan, Jianjian Wang, Jingwen Yao, Janne Estill, Shouyuan Wu, Jie Lu, Baoping Liang, Hongmin Li, Shengxin Tao, Huanli Bai, Hongliang Liu, Yaolong Chen, on behalf of COVID-19 evidence and recommendations working group

**Affiliations:** 1 Health Statistics Information Center of Health Commission of Gansu Province, Lanzhou, China; 2 School of Public Health, Lanzhou University, Lanzhou, China; 3 WHO Collaborating Centre for Guideline Implementation and Knowledge Translation, Lanzhou, China; 4 Key Laboratory of Evidence Based Medicine and Knowledge Translation of Gansu Province, Lanzhou University, Lanzhou, China; 5 Institute of Global Health, University of Geneva, Geneva, Switzerland; 6 Institute of Mathematical Statistics and Actuarial Science, University of Bern, Bern, Switzerland

**Keywords:** Corona virus disease 2019 (COVID-19), Gansu Province National Health Information Platform, Big data

## Abstract

**Background:**

In December 2019, a pneumonia caused by SARS-CoV-2 emerged in Wuhan, China and has rapidly spread around the world since then. This study is to explore the patient characteristics and transmission chains of COVID-19 in the population of Gansu province, and support decision-making.

**Methods:**

We collected data from Gansu Province National Health Information Platform. A cross-sectional study was conducted, including patients with COVID-19 confirmed between January 23 and February 6, 2020, and analyzed the gender and age of the patients. We also described the incubation period, consultation time and sources of infection in the cases, and calculated the secondary cases that occurred within Gansu for each imported case.

**Results:**

We found thirty-six (53.7%) of the patients were women and thirty-one (46.3%) men, and the median ages were 40 (IQR 31–53) years. Twenty-eight (41.8%) of the 67 cases had a history of direct exposure in Wuhan. Twenty-five (52.2%) cases came from ten families, and we found no clear reports of modes of transmission other than family clusters. The largest number of secondary cases linked to a single source was nine.

**Conclusion:**

More women than men were diagnosed with COVID-19 in Gansu Province. Although the age range of confirmed cases of COVID-19 in Gansu Province covered almost all age groups, most patients with confirmed COVID-19 tend to be middle aged persons. The most common suspected mode of transmission was through family cluster. Gansu and other settings worldwide should continue to strengthen the utilization of big data in epidemic control.

## Background

The first cases of corona virus disease 2019 (COVID-19) appeared in Wuhan, Hubei Province, China, in December 2019, and has since then spread quickly within Wuhan, Hubei, China, and the rest of the world [1, 2]. The World Health Organization (WHO) officially named the virus that caused the pneumonia epidemic in Wuhan first as “2019 New Coronavirus” (2019-nCoV) on January 12, 2020 [3] and later as “SARS Coronavirus 2” (SARS-CoV-2). The disease was officially named as COVID-19 on February 11, 2020 [4]. SARS-CoV-2 is a new type of coronavirus that has not been found in humans before. SARS-CoV-2 is characterized by its rapid spread, high contagiousness, and the high susceptibility of the population [5]. The National Health and Health Commission has reported 80,270 cumulative confirmed cases and 2981 deaths in China until 24:00 on March 3, 2020 [6], including 91 confirmed cases and two deaths in Gansu Province [7].

Big data is defined as “high-volume, high-velocity and/or high-variety information assets that demand cost-effective, innovative forms of information processing that enable enhanced insight, decision making, and process automation.” [8] In medicine, big data is increasingly used in public health promotion (disease monitoring and population management), healthcare management (quality control and performance measurement), drug and medical device surveillance, routine clinical practice (risk prediction, diagnosis accuracy, and decision support), and research [9]. The combined use of big data resources and new technologies has great potential to solve many existing medical problems and provide better evidence for decision making [10]. Big data may be one of the most efficient tools of scientific prevention and control for this public health emergency [11, 12]. Therefore, we aimed to obtain the epidemic status and characteristics of COVID-19 in Gansu Province, based on the Gansu Province National Health Information Platform, to accumulate the knowledge of this new disease and support epidemic prevention and control in Gansu and elsewhere. We present the following article in accordance with the STROBE guideline (https://www.strobe-statement.org/index.php?id=available-checklists).

## Methods

### Study design and data sources

**Fig. 1:**
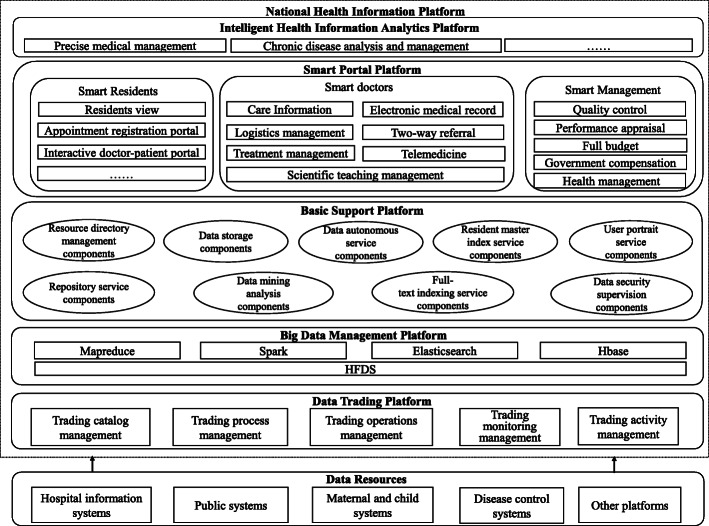
System architecture of the Gansu Province National Health Information Platform

**Fig. 2:**
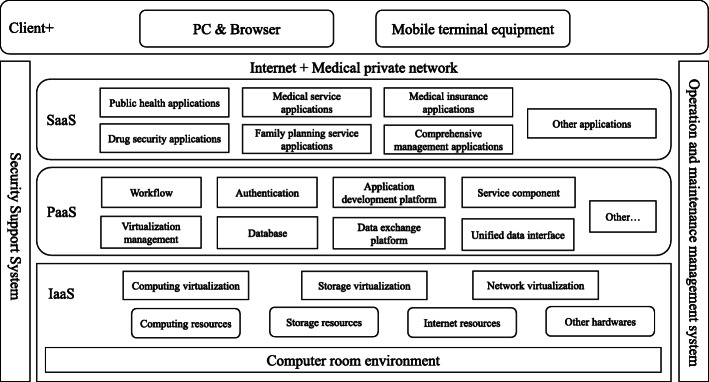
System deployment of the Gansu Province National Health Information Platform

We conducted a cross-sectional study, and obtained data from the Gansu Province National Health Information Platform and the COVID-19 epidemic supervision platform. In May 2016, the Health Commission of Gansu Province launched the construction of the National Health Information Platform, which includes the following databases: the database of the whole population (basic information collection system for the 26 million permanent residents and 3 million floating population in Gansu Province), the electronic health record database (a total of 28,953,500 electronic health records) and the electronic case database (about 110 million health records daily). It uses the data from the hospital information system above level 2, public health system, maternal and child system, disease control system and other platforms. The system architecture is shown in Fig. 1, and the system deployment is shown in Fig. 2. After 3 years of development, Gansu Province has made remarkable progress in health informatization, and accumulated a large number of high-quality health care data together.

The construction of the COVID-19 epidemic supervision platform relies on the information database containing the entire population. We wrote dynamic SQL (Structured Query Language) execution scripts, and performed data correlation, quality control, and statistics on Hadoop and Hase calculation engines. The SQL is a powerful tool for interacting with relational database systems. It enables the user to concoct complex and powerful queries in a straightforward manner, allowing sophisticated data analysis using simple syntax and structure [13]. Furthermore, we adopted implemented data quality control using multiple verification in a three-in-one manner.

### Sample size and ethical consideration

The calculation of sample size of this study is not applicable, because we used the private network of Gansu Province National Health Information Platform to obtain the relevant information of all patients with a confirmed SARS-CoV-2 infection within a 14-day period of January 23 to February 6, 2020 (the period corresponding to the estimated maximum incubation period of SARS-CoV-2, starting on the day of the first travel restrictions concerning Wuhan) [14]. The data on exposure and likely route of SARS-CoV-2 of the patients of these patients were obtained from the official website of the Gansu Provincial Health and Health Committee and media releases from the city and provincial epidemic prevention and joint control working group office. In the National Health Information Platform, all operations are strictly done in accordance with the outline of *Action plan for big data development* [15] and strictly comply with the personal information protection law and ethical standards. Our data was obtained with the consent of the Health Commission of Gansu Province. To protect the privacy of the individuals, data encryption technology is used in occasion of data use, processing, sharing and interaction. None of the study personnel could see the personal information of individuals.

### Inclusion and exclusion criteria

We defined a confirmed case of COVID-19 according to the “Diagnosis and treatment of COVID-19 (Trial Version 3)” [16]. For a case to be confirmed as COVID-19, the detection of either SARS-CoV-2 in a real-time fluorescent PT-PCR of respiratory specimens or blood specimens, or a gene highly homologous with the known gene of SARS-CoV-2 in viral gene sequencing, was required.

In this study, we excluded patients who were suspected cases, but the results of nucleic acid detection were negative.

### Data extraction and analysis

Descriptive statistical analyses were performed. Data on basic case information such as age, gender, history of exposure, incubation period, and treatment time were collected from the Gansu Province National Health Information Platform. Continuous variables were expressed as medians, and categorical variables as absolute numbers and percentages, and all these statistical analyses were performed with the Statistical Program for Social Sciences SPSS 25.0 software (SPSS Inc., Chicago, Illinois, USA).

In addition, we analyzed the possible source of infection among the confirmed cases in Gansu Province through collecting data on possible routes of exposure and following up the patient, this was conducted by the private network of Gansu Province National Health Information Platform. We also explored the possibility of having a “super spreader”, defined as an individual who infected more than 10 persons [17], linked to the cases in Gansu.

## Results

### The basic characteristics of the cases

We retrieved data from 67 cases of COVID-19 confirmed between January 23 and February 6, 2020. Thirty-six (53.7%) of the patients were women and thirty-one (46.3%) men. And the median ages were 40 (IQR 31–53) years.

Of the 67 cases of COVID-19 confirmed between January 23 and February 6, 2020, forty-one (61.2%) had spent time outside Gansu province, and 28 cases (41.8%) in Wuhan. The time interval from arrival into Gansu Province to the onset of symptoms ranged between 8 days before arriving in Gansu to 12 days after arriving in Gansu, with a mean of 3.5±4.7 days after arrival. The interval from onset of symptoms to the initiation of treatment was 0–18 days (mean 4.4±4.0 days), from onset of symptoms to hospitalization 0–17 days (mean 6.4±3.7 days). In addition, 35 (52.2%) of the confirmed cases come from only ten families, showing obvious family clustering.

### Source of infection

**Fig. 3:**
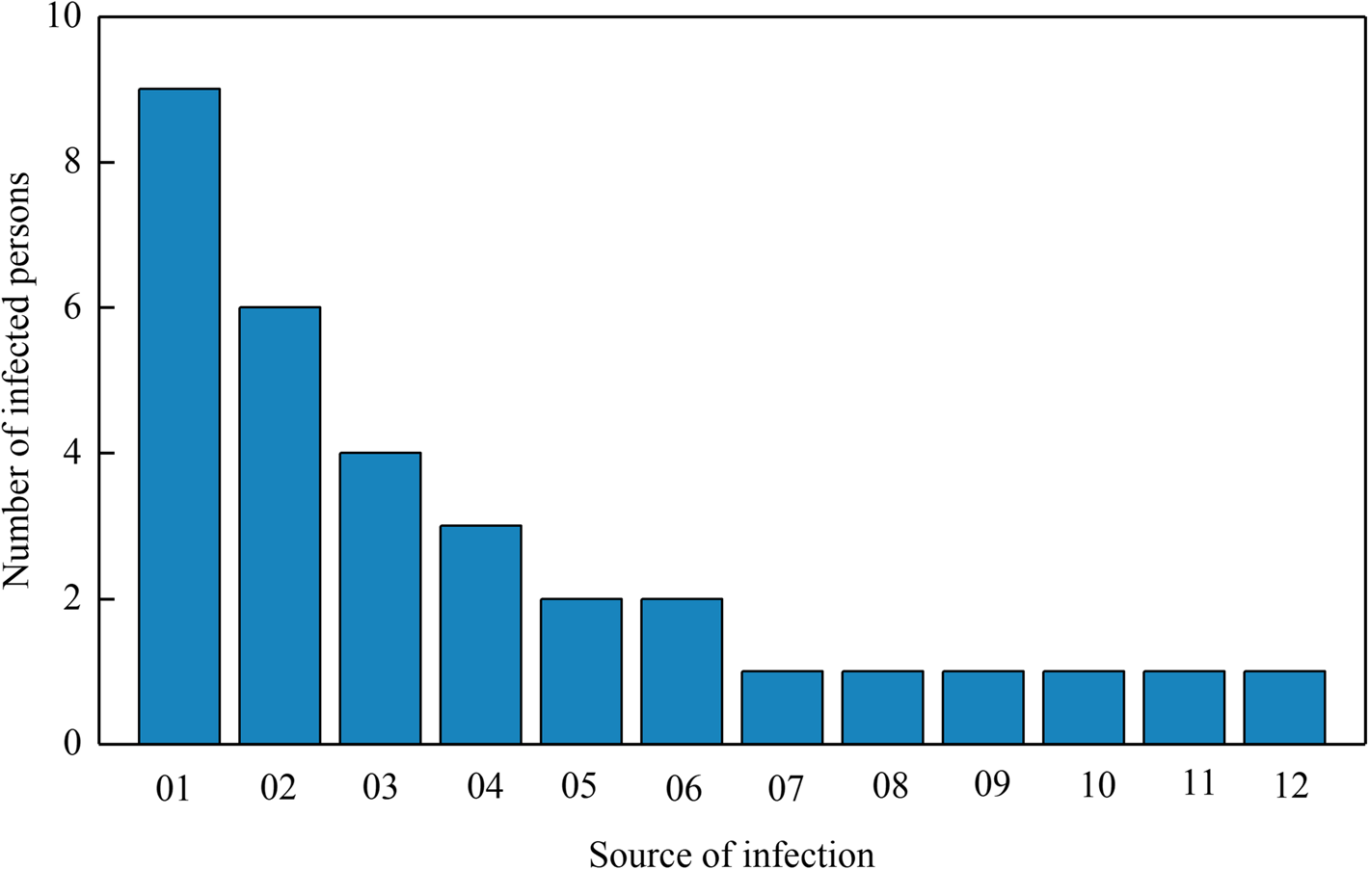
Number of secondary cases linked to the twelve sources who infected at least one other individual

We traced the epidemiological history of the 67 confirmed cases, and found that 35 infections were directly imported from outside the province or from an unknown source. Twenty-three of these patients were properly controlled and caused no further transmissions. Twelve patients continued to spread the infection further, causing therefore 32 onward transmissions. The majority (*n*=19) of these 32 patients were linked to only three sources. This calculation shows that none of the patients from Gansu fulfilled the definition of a “super spreader”. (Fig. 3).

## Discussion

This is the first “big data” study to explore the transmission chains and epidemiological characteristics of COVID-19 in Gansu Province. We found more women than men were diagnosed with COVID-19, and most patients with confirmed COVID-19 tend to be middle aged persons, covering the duration of the estimated maximum incubation period of SARS-CoV-2 starting from 23 January 2020 when the first travel restrictions took place. Most infections in Gansu Province were imported from Wuhan. The most common suspected mode of transmission was through family clusters, and we found no clear reports of other modes of transmission.

### Gender, age and aggregation

More women than men were diagnosed with COVID-19 in Gansu Province. This finding is inconsistent with the situation at the beginning of the outbreak, men accounting for 56% of the first 425 patients diagnosed in Wuhan [18]. A retrospective, single-center study published on February 15, 2020 on 99 cases in Wuhan, also indicated that men were at higher risk than women. The proposed reduced susceptibility of females to viral infections could be attributed to the protection from the X chromosome and sex hormones, which play an important role in innate and adaptive immunity [19, 20]. However, there is no clear evidence yet for the higher susceptibility of women than men.

Although most patients with confirmed COVID-19 tend to be middle aged persons, the age range of confirmed cases of COVID-19 in Gansu Province covered almost all age groups. This is in line with previous research findings. People of all ages have been shown to be susceptible to SARS-CoV-2, and are thus at risk of acquiring the infection as long as the conditions necessary for transmission are met [5, 21]. An analysis of 4021 confirmed cases in China also showed that people of all ages are generally susceptible: 71.5% of the patients were aged 30 to 65 years, and 0.4% were children under the age of 10 years [22]. However, the risk of acquiring SARS-CoV-2 may be increased in the elderly and people with chronic underlying diseases such as asthma, diabetes and heart disease [21].

At the early stage of the epidemic, cases of COVID-19 were mainly sporadic. The proportion of clustered epidemics in various locations has continued to increase, which has also changed the development of the epidemic and the sources of exposure. The number of cases linked to clustered epidemics is estimated to account for 50 to 80% of all confirmed cases in several provinces and cities including Beijing, Shanghai, Jiangsu, and Shandong [21]. The results of this study, covering a period of 14 days which is the estimated maximal incubation period, show that although no super spreaders were found, many cases were clustered in families or neighborhoods. This shows that there was ongoing human-to-human transmission also in Gansu Province. These characteristic related to clustering and sources of infection are consistent with reports from Shanxi, Chongqing, and other provinces and cities [23, 24]. The incubation period of SARS-CoV-2 is generally 4 to 7 days, and a large number of suspected patients and asymptomatic infections become the main source of infection [20, 21]. Therefore, it is important to track people with asymptomatic infections, and block the occurrence of familial and spacious aggregation. Gansu Province National Health Information Platform is linked with the entire population information database. Such data sources give new opportunities to accurately delineate the group of people with closest contacts and realize early warning to prevent family aggregation of the disease.

### The potential of big data on prevention and control

As a new infectious disease, the spread of COVID-19 was accelerated at the beginning of the epidemic by the delayed in diagnosis, treatment, and epidemic management due to lack of awareness. China’s traditional surveillance network mainly relies on reporting and summarizing the situation, which is however too slow to meet the need for rapid response to the epidemic. Big data has a huge potential to help to follow, control and respond to epidemics rapidly. The use of information technology and big data as an effective auxiliary method for epidemiological investigations can not only achieve early detection, early reporting, early isolation, and early treatment of cases, but also quickly map out the current status of the disease, grasp the patients’ past medical history, and help to track the sources of infection and control the epidemic. As the big data network allows almost real-time disease monitoring, this comprehensive and rapid surveillance method will make public health surveillance more sensitive, especially to trace the unconscious close contacts and provide the necessary control measures to prevent further infections [25].

Big data in the field of medicine and public health has become one of the most important medical resources, and has played an active role in the prevention and control of COVID-19 in Gansu Province. In particular, it has provided great support for the management of source of infection and the development of epidemiological investigations. In fact, many provinces in China have built and are constantly optimizing their own big data platforms, but the regional platforms are not yet well connected [26]. In the current Chinese medical system, it is almost impossible to track a patient through electronic record systems for clinical purposes as there is no unified national platform that can consolidate all the data from all healthcare institutions in China [27]. Therefore, we only included COVID-19 confirmed cases from Gansu Province for analysis, and could not integrate data from all big data platforms in China for a larger analysis, which is also the limitation of our research. In the next step of prevention and control, Gansu and other provinces in China should continue to strengthen the innovation construction of “big data + epidemiology” [25], prevent the recurrence of imported cases and cluster epidemics, and continuously improve the construction and promotion of big data related platforms, so as to provide a theoretical basis for facing related emergencies to facilitate scientific epidemic prevention and decision-making in the future.

## Conclusion

In summary, women formed the majority of COVID-19 cases in Gansu during the two-week observation period. The overall age range was broad, confirming that people of all ages are susceptible to the virus. The most common suspected mode of transmission was through family cluster, which is promising since it makes the tracing of close contacts easier. This study is the first exploratory application of the Gansu Province National Health Information Platform for the prevention and control of COVID-19. We will continue to follow up and investigate the patients with COVID-19 in Gansu province through the platform, and further explore the second generation incidence rate, risk assessment and long-term effect of COVID-19. The results of this study demonstrate the great potential of “big data” in epidemiological research. Gansu and other provinces and settings should continue to strengthen the utilization of “big data” in epidemic prevention and control, to prevent the development of imported cases into clustered epidemics.

## Data Availability

The datasets generated and analysed during the current study are not publicly available due it is from a private network of Gansu Province National Health Information Platform, but are available from the corresponding author on reasonable request.

## Abbreviations

COVID-19: Corona virus disease 2019
WHO: World Health Organization
2019-nCoV: 2019 New Coronavirus
SARS-CoV-2: SARS Coronavirus 2
SQL: Structured Query Language
